# Artificial Intelligence Meets Item Analysis (AI meets IA): A Study of Chatbot Training and Performance in detecting and correcting MCQ Flaws

**DOI:** 10.12669/pjms.41.3.11224

**Published:** 2025-03

**Authors:** Mashaal Sabqat, Rehan Ahmed Khan, Masood Jawaid, Madiha Sajjad

**Affiliations:** 1 Mashaal Sabqat, MBBS, MHPE Assistant Professor Medical Education, Islamic International Medical College, Assistant Director Riphah Institute of Assessment, Riphah International University, Islamabad, Pakistan; 2 Rehan Ahmed Khan, MBBS, MHPE, FCPS, FRCS (Surgery), PhD ME Dean Riphah Institute of Assessment, Riphah International University, Islamabad, Pakistan. Professor of Surgery, Department of Surgery, Riphah International University, Rawalpindi, Pakistan; 3 Masood Jawaid, MBBS, MHPE, MCPS, MRCS (Glasg), FCPS (Surgery) Associate Editor Pakistan Journal of Medical Sciences. Director Medical Affairs, PharmEvo (Private) Ltd., Karachi, Pakistan; 4 Madiha Sajjad, MBBS, MHPE, FCPS (Pathology) Professor, Histopathology, Islamic International Medical College, Riphah International University, Islamabad, Pakistan

**Keywords:** Artificial Intelligence, Item analysis, Multiple-choice questions

## Abstract

**Objective:**

To explore the potential of AI-powered chatbots, specifically ChatGPT, in identifying and correcting flaws in MCQs.

**Methods:**

A three-phase-Interventional study was conducted from February to August 2023 at Riphah International University, Islamabad. In Phase-1, flawed MCQs were selected from the NBME guide and fed into ChatGPT. ChatGPT identified item flaws and suggested corrections. In Phase-2, ChatGPT was trained to detect flaws in MCQs with text data from the NBME item writing guide. In Phase-3, ChatGPT was again tested to detect flaws and correct MCQs. Data were analyzed using SPSS, Version 26 and presented using percentages and McNemar’s test with exact conditional method.

**Results:**

ChatGPT could identify and correct flaws such as use of “None of the above,” “Grammatical cues,” “absolute terms,” and “inconsistently presented numerical data.” However, it struggled with flaws related to “complicated stems,” “long or complex options,” and “vague frequency terms.” After training, ChatGPT became better at identifying and correcting flaws related to complicated stems and absolute terms. It also struggled with recognizing “nonparallel options,” “convergence,” and “word repetition,” both before and after training. ChatGPT’s performance deteriorated during peak hours. The test of significance showed no measurable increase in ChatGPT’s efficiency in detecting item flaws (p = 1.00) and correcting them (p = 0.125).

**Conclusion:**

AI is revolutionizing industries and improving efficiency, but limitations exist in complex conversations, analysis, accuracy, and error prevention. Ongoing research is vital to unlocking AI’s potential, especially in education.

## INTRODUCTION

Multiple choice questions (MCQs) are a widely used assessment tool in various domains, including medical education.^1^ However, creating well-designed multiple-choice questions can be a challenging task that demands meticulous attention to several elements, including the effectiveness of distractors, the complexity of the question, and the cognitive level required.^2^,^3^ To create high-quality written exam questions for the Health Sciences, the National Board of Medical Education (NBME) Item Writing Guide^4^ is an invaluable resource that can be downloaded for free. MCQs with poorly worded stems or ambiguous answer options can negatively impact the reliability and validity of test results, which can ultimately lead to inaccurate and unreliable assessments. Identifying item flaws is often a time-consuming and laborious process that may require significant input from faculty members.^5^

Recent advances in the fields of artificial intelligence (AI), natural language processing (NLP) and machine learning (ML) have led to the development of chatbots, for example, Rose, Mitsuku, ChatGPT, Xiaoice and Replika. These chatbots are automated conversational agents that offer benefits like real-time responsiveness, 24/7 availability, personalized responses, and cost effectiveness.^6^,^7^ This has led to their growing usage in various fields, including diverse educational contexts.^8^ Several studies have highlighted their utility in different areas of educational assessment.^9^ However, the effectiveness of their use in detecting item flaws in MCQs is yet to be explored.^10^,^11^ This gap in knowledge highlights the need for further research to explore how efficiently chatbots can detect and correct the item flaws in MCQs, and if they may be trained to do so according to best-evidence guidelines.

ChatGPT, developed by OpenAI, is a well-known AI-powered chatbot built on the GPT (Generative Pre-trained Transformer) technology. This allows it to interact with users in a conversational manner, to intelligently answer follow-up questions, admit its mistakes and challenge incorrect premises, while retaining its accumulated knowledge and generalization ability gained from pre-training.^12^,^13^ In this study, we aimed to investigate the ability of chatbots, specifically ChatGPT, in identifying and correcting item flaws in multiple-choice questions (MCQs) used in medical education. We also hope to assess the effectiveness of training it to do this, according to the NBME guidelines.

Therefore, the objectives of this study are:

- To explore the ability of ChatGPT, in detecting and correcting item flaws in MCQs.
- To investigate the potential of training ChatGPT using the NBME guidelines to detect and correct flaws in MCQs according to best-evidence guidelines.

Should chatbots prove to be a successful tool for identifying flaws in MCQs, they hold the potential to expedite and streamline this process, reducing the workload of the faculty.^14^ Furthermore, the ability of chatbots to analyze large amounts of data can enable them to identify patterns and trends that may be difficult to detect manually. Hence, the use of chatbots like ChatGPT for MCQs item flaw detection has the potential to not only save time and effort but also enhance the accuracy and consistency of the process.

## METHODS

This is a three-phase-Interventional design study. We used the latest free version of ChatGPT available at the time of the study (Free Research Preview, GPT-3.5, November 2022)^15^ to explore the chatbot’s effectiveness in detecting and correcting flaws in MCQs.

### Ethical Approval:

The study was approved by ethical committee from Riphah International University, Islamabad (IRC/23/3049, February 28, 2023). The study was carried out between February to August 2023.

### PHASE 1- Pre-Training:

Flawed A-type MCQs (one-best-answer format) were selected from the NBME item writing guide and put into ChatGPT. A-type MCQs are widely used in medical education. One MCQ was chosen from each of the 13 flaw categories outlined in the guide to assess the chatbot’s ability to identify and correct item flaws. Table-I details these flaw categories.

**Table-I:** Categories of item flaws to be tested for detection by ChatGPT.

Flaws related to irrelevant difficulty	
1.	Long or Complex Options
2.	Numeric Data Presented Inconsistently
3.	Vague terms
4.	‘None of the above’
5.	Nonparallel options
6.	Complicated stems
7.	Negatively Phrased Lead-ins
***Flaws that cue the testwise examinee***	
8.	Grammatical Cues
9.	Grouped or Collectively Exhaustive Options
10.	Absolute Terms
11.	Correct Option Stands Out
12.	Word Repetition (Clang Clues)
13.	Convergence

To instruct ChatGPT to identify item flaws and suggest corrections, we developed the following initial prompt:

### Initial prompt:

*“Can you review the following A-type (One-best-answer) MCQs to detect item flaws in them? Also give the corrected version of each MCQ.”*

The prompt was refined collaboratively through prompt engineering techniques, including clear and specific instructions, explicit constraints, and experimenting with context and examples.^16^ It was further fine-tuned through iterative testing.^16^ (Additional file 1) The final prompt incorporated a Zero-shot-CoT approach by adding ‘Let’s think step by step’ to promote structured reasoning and enhance response quality.^17^,^18^

### Final prompt:

*“Can you review all parts (stem, lead-in, options) of the following A-type (One-best-answer item) MCQ? Detect any flaws (Issues related to irrelevant difficulty, Cues to the test-wise examinee) if present, and explain the flaws. Also, give the corrected version of the MCQ by removing/ correcting the detected flaws. ‘Let’s think step by step’.”*

Each flawed MCQ was entered into ChatGPT preceded by the final prompt in a separate window to stay within the model’s token limit and minimize memory retention bias.^19^

### (Additional file 2) PHASE-2 - Training the ChatGPT:

We trained ChatGPT to detect item flaws in MCQs using best-evidence guidelines by providing text data for each item flaw (Table-I) and the five basic rules for constructing one-best-answer type MCQs from the NBME guide (Additional file 3). The training text was introduced with the following prompt, refined through iterative testing and consensus among the authors: *‘I am telling you some best-evidence guidelines for writing one-best type MCQs, along with item flaws, so you can detect flaws in one-best-type MCQs that I will provide you later.’* PHASE-3 - Post-Training: Flawed MCQs from Phase 1 were revisited, with ChatGPT tasked to detect flaws and suggest corrections using the same final prompt employed in Phase 1. Mirroring the conditions of Phase 1, a separate window was used for each flawed MCQ. Phases 2 & 3 were conducted concomitantly for each entry into the ChatGPT interface. In each window, we first input the Phase-2 prompt along with training text data, followed by the Phase-3 prompt and the flawed MCQ. Additionally, supplementary prompts were introduced to enhance ChatGPT’s training and optimize responses. A flexible strategy was used for administering these supplementary prompts, allowing for real-time adjustments targeting specific flaws to guide the chatbot towards the desired output. The number of additional prompts addressing each flaw was limited to two to ensure procedural consistency (Additional file 4).

### Data analysis:

Data were analyzed using the Statistical Package for the Social Sciences (SPSS), Version 26. We determined the frequency of item flaw detection and correction by ChatGPT, both before and after text data training (see Additional files 5 & 6 for data sets). Given the paired nominal nature of our data and the presence of zero counts in one or more cells within the contingency tables, we opted for McNemar’s test using the exact conditional method to evaluate the significance of differences in ChatGPT’s performance pre- and post-training,^20^ The confidence interval was set at 95%, under the null hypothesis that there is no significant difference in the ability of ChatGPT to identify and correct item flaws before and after training. A p-value of less than 0.05 was predetermined as the threshold for statistical significance, allowing for rejection of null hypothesis if met.

## RESULTS

**Fig.1:**
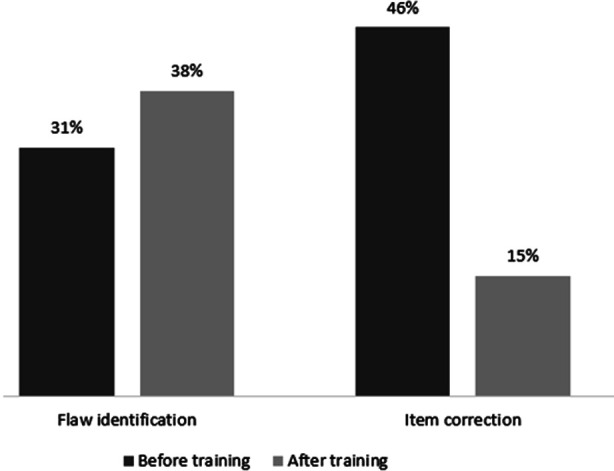
Percentages of item flaw identification and correction by ChatGPT before and after text data training.

The results showed variable responses from ChatGPT towards different item flaws. Table-II shows their summary. Percentages of these responses before and after training is shown in Fig-1. The analysis showed no significant improvement in ChatGPT’s ability to detect or correct item flaws after training (p-values: 1.00 for identification, 0.125 for correction).

**Table-II:** Summary of Item flaw detection and correction by ChatGPT.

Item flaws		Identification by ChatGPT		Correction by ChatGPT	
No.	Categories	Before training	After training	Before training	After training
1	Long or Complex Options	-	-	-	-
2	Numeric Data Presented Inconsistently	+	+	-	-
3	Vague frequency terms	-	+	-	-
4	‘None of the above’	+	+	+	-
5	Nonparallel options	-	-	-	-
6	Complicated stems	-	-	+	-
7	Negatively Phrased Lead-ins	-	-	+	-
8	Grammatical Cues	+	+	+	+
9	Grouped or Collectively Exhaustive Options	-	-	-	-
10	Absolute Terms	+	+	+	-
11	Correct Option Stands Out	-	-	+	+
12	Word Repetition (Clang Clues)	-	-	-	-
13	Convergence	-	-	-	-

For question stems, ChatGPT initially mislabeled “complicated stems” as “incomplete” and failed to simplify effectively, resulting in MCQs with incorrect structure and implausible information. Post-training, it identified and simplified stems better but complicated the answer options. ChatGPT could identify flaws like “None of the above,” “grammatical cues,” and “absolute terms” before training. However, after training, it missed the “None of the above” flaw until prompted, although it consistently addressed grammatical cues and absolute terms. It did not recognize “long or complex options” as a flaw before or after training, disputing the subjective perception of complexity. When prompted, revisions were minimal.

Furthermore, ChatGPT identified “inconsistently presented numerical data” as “vague options” without prior training but struggled to correct it. For example, given options like “Less than 20%, 20 to 30%, Greater than 50%, 75%, 90%,” it revised them to “Less than 5%, 5-10%, 10-20%, 20-30%, Greater than 30%,” failing to achieve the necessary consistency. Even after text data training and three additional prompts, ChatGPT continued to struggle with consistency, adjusting the same options to “less than 10%, 10-20%, 20-30%, 50-60%, and 85-90%.” For “ambiguous frequency terms,” ChatGPT recognized the flaw post-training, though mislabeling it as “absolute terms.” The terms remained vague in the revised question, which also featured non-uniform options. It also failed to detect flaws such as “nonparallel options,” “convergence,” and “word repetition” before and after training, considering the MCQs valid.

Pre-training, ChatGPT did not recognize “negatively phrased lead-ins” as flaws, but post-training identified them, replacing them with another negative phrase that required further prompting. Initially, ChatGPT did not recognize “grouped or collectively exhaustive options” as a flaw in the given MCQ. To generate a corrected MCQ without such options, it required five additional explanations and an example, in addition to the initial text training data. Subsequently, ChatGPT began incorrectly identify “collectively exhaustive options” as flaws in some MCQs, even when they were not applicable. Lastly, before training, ChatGPT overlooked “long correct options” but produced more balanced options post-training. During peak hours, ChatGPT’s performance declined, forgetting instructions and requiring repeated inputs.

## DISCUSSION

The purpose of this study was to investigate the potential of chatbots, particularly ChatGPT, in detecting and correcting item flaws in MCQs. Findings suggest that while ChatGPT can identify and correct certain types of item flaws such as grammatical cues, absolute terms, and extraneous information, its overall effectiveness in detecting and correcting technical item flaws remained questionable, as it was unable to identify and correct many item flaws even after text data training. The findings of the study likely stem from the ChatGPT’s limitations in analysing and applying complex principles. Gonda et al.^21^ and Bang et al.^22^ have noted similar weakness in chatbots’ ability to handle complex conversations and reasoning abilities. Another contributing factor may be the extensive data on which ChatGPT is trained, which influences its responses.^23^ Our results indicate that, although ChatGPT is designed as a conversational agent, it lacks the ability to learn new information in real time. The flaws effectively identified and corrected by ChatGPT were predominantly language-related, aligning with its nature as a language model.

The study found that ongoing human input and supervision are necessary for the effective detection and correction of flaws in MCQs using ChatGPT, consistent with the observations of Kasneci et al.^24^ However, the practicality of employing ChatGPT for this task is limited due to the considerable time and effort required, which may be equivalent to or greater than the time needed for manual correction of MCQs. In this regard, Sam Altman, CEO of OpenAI, cautioned against relying on ChatGPT for important tasks.^12^ The study highlighted that ChatGPT-generated corrected MCQs may contain nonsensical language and implausible options, an issue echoed by Stephen Atlas and observed in our research.

The findings raise concerns regarding automation bias, which occurs when users place undue trust in automated systems like AI, often overlooking errors due to a belief in their infallibility.^25^ Given the high accuracy required for flaw detection and correction in MCQs, reliance on ChatGPT could lead to the incorporation of erroneous outputs into assessments, potentially undermining educational quality and assessment standards. The observed limitations emphasize the need for critical oversight when integrating automated systems into precision-dependent tasks. This study serves as a reminder that while automated tools can assist educational processes, they should not be trusted without adequate human supervision to mitigate the risks of automation bias.

The research outcomes are expected to contribute significantly to the broader field of AI in education, helping educators understand ChatGPT’s capabilities and limitations, particularly for tasks requiring complex analyses. The study paves the way for targeted improvements in AI systems. It also recognizes chatbots’ limitations and encourages a cautious and informed approach to AI integration in education.

### Limitations:

The study has some limitations that should be noted. First, only one MCQ per flaw was used to test each of the 13 item flaw categories. Using multiple MCQs, including those not published online prior to 2022, could provide more insights into ChatGPT’s capabilities and increase the generalizability of results. Secondly, ChatGPT was trained solely on text data in this study. Future research could optimize its training by employing advanced methods, such as deep learning algorithms like the ’Transformer,’ which could enhance ChatGPT’s understanding of word relationships. Lastly, for this study, text was entered directly into the OpenAI interface. Future studies could utilize an API to connect to the OpenAI interface, increasing domain specificity. Investigating other chatbot applications in assessment, such as MCQ generation and question checking, along with evaluating different AI models for educational purposes, could also be beneficial.

## CONCLUSION

Artificial Intelligence (AI) is rapidly transforming industries by automating processes and improving efficiency. However, its limitations in handling complex conversations, and applying complex principles hinder its effectiveness in detecting and correcting MCQ flaws. The variability of ChatGPT’s performance during peak hours further challenges its reliability for critical educational tasks. Future AI advancements may overcome these limitations, making ongoing research and development essential for unlocking AI’s full potential in various fields, including education.

### Author’s Contribution:

**MS:** Conceived the idea and made a proposal and led on data collection.

**RAK**, **MJ**, and **MS:** Did data analysis.

**MS** drafted the manuscript.

**RAK**, **MJ**, and **MS:** Finalized the manuscript.

All authors finalized the manuscript before submission and are accountable for the integrity of research.
